# Optimisation of lithium-substituted bioactive glasses to tailor cell response for hard tissue repair

**DOI:** 10.1007/s10853-017-0838-7

**Published:** 2017-02-09

**Authors:** Jeison Gabriel da Silva, Rebecca Babb, Christoph Salzlechner, Paul T. Sharpe, Delia S. Brauer, Eileen Gentleman

**Affiliations:** 1 Craniofacial Development and Stem Cell Biology, King’s College London, 27th Floor, Guy’s Hospital, London, SE1 9RT UK; 2 Otto Schott Institute of Materials Research, Friedrich Schiller University Jena, 07743 Jena, Germany

## Abstract

Bioactive glasses (BG) are used clinically because they can both bond to hard tissue and release therapeutic ions that can stimulate nearby cells. Lithium has been shown to regulate the Wnt/β-catenin cell signalling pathway, which plays important roles in the formation and repair of bone and teeth. Lithium-releasing BG, therefore, have the potential to locally regulate hard tissue formation; however, their design must be tailored to induce an appropriate biological response. Here, we optimised the release of lithium from lithium-substituted BG by varying BG composition, particle size and concentration to minimise toxicity and maximise upregulation of the Wnt target gene *Axin2* in in vitro cell cultures. Our results show that we can tailor lithium release from BG over a wide therapeutic and non-toxic range. Increasing the concentration of BG in cell culture medium can induce toxicity, likely due to modulations in pH. Nevertheless, at sub-toxic concentrations, lithium released from BG can upregulate the Wnt pathway in 17IA4 cells, similarly to treatment with LiCl. Taken together, these data demonstrate that ion release from lithium-substituted BG can be tailored to maximise biological response. These data may be important in the design of BG that can regulate the Wnt/β-catenin pathway to promote hard tissue repair or regeneration.

## Introduction

Bioactive glasses (BG) have been used clinically in bone and dental restorations for more than 30 years [1, 2]. In addition to their well-described surface reactive properties, which allow them to directly bond to biological tissues, BG also dissolve in the presence of biological fluids, releasing ions that affect nearby cells. We and others have previously shown that ions released from BG can alter cell response in both targeted and global contexts [3–6]. For example, incorporating fluoride into BG upregulates markers of bone mineralisation in human osteogenic cell cultures [7], and cobalt-containing BG mimic hypoxia by stabilising hypoxia inducible factor-1α [8]. Similarly, modifying BG with strontium promotes an anabolic effect on osteoblasts and an anti-catabolic effect on osteoclasts [9, 10], and surprisingly, strongly regulates the isoprenoid pathway in human mesenchymal stem cells [11]. BG can also be incorporated into other biomaterials, such as glass ionomer-type [12, 13] and other bone cements [14], which will likewise release BG ions over time [12].

Ion release from BG is governed by a number of factors including characteristics of the BG, such as its composition, average particle size and concentration in solution, as well as characteristics of the dissolution solution, including its pH and ionic composition. Moreover, the entire process is time dependent, as degradation of the BG structure is countered by the precipitation of mineral (usually hydroxyapatite) on its surface and dynamic changes in pH. Understanding the interplay of these factors is important in BG design as excessive dissolution can result in toxicity due to the ions themselves [15] or variations in local pH [6].

Lithium has been used clinically to treat psychiatric patients for decades. However, in addition to its effects on mood stabilisation, lithium also inhibits glycogen synthase kinase-3β (GSK-3β), which upregulates canonical Wnt/β-catenin cell signalling [16]. As Wnt signalling plays important roles in mineralised tissue formation [17, 18], it is not surprising that lithium treatment has been shown to enhance bone formation via this pathway in mice [19]. And, in accordance with these observations, patients on long-term lithium therapy show enhanced bone mineral density [20]. Moreover, lithium also appears to affect the formation of dental tissues. Lithium chloride topically applied to the dental pulp promotes dentine regeneration [21] and administered to neural crest cells in vitro induces odontoblast differentiation by activating Wnt signalling [22]. Lithium can be incorporated into BG by replacing the alkali sodium ion with lithium, which in addition to potentially providing a local therapeutic effect via regulation of Wnt signalling, also reduces the BG’s crystallisation tendency, making it more amenable to scaffold preparation [23, 24]. These BG show reduced ion release and slightly delayed apatite formation in vitro compared to BG without lithium, but apatite formation is not dramatically reduced [23, 24]. Miguez-Pacheco et al. [25] showed that lithium-containing BG can release therapeutic levels of lithium ions, and Khorami et al. [26] demonstrated that they stimulate rat calvarial osteoblast proliferation and alkaline phosphatase activity in a dose-dependent manner.

The concentration of lithium in the serum of patients treated with lithium has been reported in the range 0.8 mM (5.5 ppm) [20], and so this level has been suggested to be in the therapeutic range for targeting bone formation via activation of the Wnt pathway. However, in vitro studies suggest 20 mM LiCl (139 ppm) is necessary to regulate the Wnt pathway in mouse calvarial osteoblasts [19]. When released from lithium-substituted BG, 17 ppm lithium has been reported to promote the cementogenic differentiation of periodontal ligament cells via activation of the Wnt pathway [27]. Nevertheless, the appropriate concentration of lithium necessary to be released from a BG to stimulate Wnt signalling and promote mineralised tissue formation is still controversial. Here, we aimed to systematically explore how to tailor the ionic release of lithium from lithium-substituted BG and lithium-substituted BG-doped GIC to ensure their biocompatibility and maximise their effect on mineralised tissue formation via Wnt pathway activation.

## Materials and methods

### BG synthesis

SiO_2_–P_2_O_5_–CaO–Na_2_O–Li_2_O BG were formed using a melt quench route, as previously described [23]. Briefly, varying amounts of Li_2_O were substituted for Na_2_O on a molar basis (Table 1) such that BG structure (i.e. silicate network polymerisation) did not change with the incorporation of lithium. The components were melted in a platinum crucible at 1250 °C for 1 h, then at 1350 °C for 1 h and rapidly quenched in water to obtain glass frit. BG frit were crushed in a steel mortar and sieved to particle sizes of either 0.1–1.0 mm (large) or <38 μm (small), to vary relative BG surface area.

**Table 1:** Glass compositions (mol %)

	SiO_2_	P_2_O_2_	CaO	Na_2_O	Li_2_O
Li0 (45S5)	46.1	2.6	26.9	24.4	–
Li100	46.1	2.6	26.9	–	24.4
Li75	46.1	2.6	26.9	6.1	18.3
Li50	46.1	2.6	26.9	12.2	12.2
Li25	46.1	2.6	26.9	18.3	6.1

### Formation of glass ionomer cements (GIC)

GIC modified with lithium-substituted BG were formed by replacing part of the powder phase of Ketac™ Cem radiopaque (3M) GIC with <38 µm BG particles (small). To maintain a constant molar amount across the different BG compositions and to account for differences in the atomic weights of lithium and sodium, the mass of substituted BG was adjusted according to Table 2. GIC were mixed according to the manufacturer’s instructions using 50 µL of liquid per 315 mg of powder. Once thoroughly mixed, the cement was placed in pre-sterilised moulds measuring 6 mm in diameter and 6 mm in height and placed at 37 °C for 1 h to set.

**Table 2:** Amount of BG and GIC powder (in g; accounting for differences in atomic weights of sodium and lithium) incorporated into Ketac™ Cem Radiopaque to create lithium-releasing GIC

	Molar equivalent to 1 g 45S5 (g)	Ketac™ Cem Radiopaque (g)
Li0 (45S5)	1	9
Li25	0.968	9.032
Li50	0.936	9.063
Li75	0.904	9.095
Li100	0.873	9.126

### Cell culture

The mouse osteoblast cell line MC3T3-E1 was obtained from the European Collection of Cell Cultures (Salisbury, UK) and cultured under standard conditions (37 °C, 5% CO_2_/95% air, 100% humidity) in alpha minimum essential medium (αMEM) supplemented with 10% (v/v) foetal bovine serum (FBS) and 2 mM l-glutamine (all from Life Technologies, Paisley, UK). The mouse dental pulp cell line 17IA4 [28] was cultured under standard conditions in αMEM supplemented with 10% (v/v) FBS and 2 mM l-glutamine (all from Life Technologies, Paisley, UK).

### Preparation of BG-conditioned cell culture medium

To create the initial conditioned medium (referred to as 1×), 6 mg of large BG particles per mL αMEM was placed in 50 mL centrifuge tubes and placed onto a laboratory tube roller at 37 °C. After the specified time period, BG particles were removed with a 0.22-μm syringe filter and the media were stored at 4 °C prior to use. 10× medium and 50× medium were created similarly except the concentration of BG in the media was increased to 60 mg/mL and 300 mg/mL, respectively. Small BG particles at equivalent mass to volume ratios were used to make conditioned medium when specified. Measurements of cell culture medium pH were made using a standard pH metre.

### ISO10993:5 cytotoxicity testing

Cytotoxicity testing of BG-conditioned cell culture medium and GIC was carried out according to a modified version of ISO10993:5, as previously described [11]. Organotin-stabilised polyvinyl chloride (PVC) sheet and non-toxic (Med7536 noDop) tubing (both kindly provided by Raumedic AG, Helmbrechts, Germany) were used as positive and negative controls, respectively. Briefly, to create control medium, materials were sterilised with 70% ethanol for 1 h, washed with phosphate buffered saline (PBS) and then soaked in αMEM at a surface area to volume ratio of 3 cm^2^/mL for 7 days at 37 °C. GIC-conditioned medium was created identically with the omission of the sterilisation step (as cements were formed under sterile conditions). Conditioned media were used neat or diluted 1:1 with fresh αMEM (not soaked with BG) when noted. Standards were also created by adding LiCl (Sigma) to cell culture media. MC3T3-E1 were plated in 96-well plates at 20000 cells/cm^2^ and allowed to attach for 24 h. Standard culture medium was then replaced with LiCl-containing, conditioned or control media supplemented with 10% FBS and 2 mM l-glutamine for 24 h. To determine cell metabolic activity, 20 μL of a 5 mg/ml solution of MTT [3-(4,5-dimethylthiazol-2-yl)-2,5-diphenyltetrazolium bromide; Sigma-Aldrich, Dorset, UK] in PBS was added to each well and returned to the incubator for 4 h. The resulting formazan product was dissolved in 200 μL of dimethyl sulfoxide (Sigma-Aldrich, Dorset, UK), and the absorbance of the product was measured on a colorimetric plate reader (Thermo Multiskan Ascent 354 microplate reader) at 540 nm.

### Gene expression analyses

17IA4 were plated in 12-well plates at 25000 cells/cm^2^ and allowed to attach for 24 h. Standard culture medium was then replaced with conditioned or control medium supplemented with 10% FBS and 2 mM l-glutamine for 24 h. Cells were detached with trypsin, and pellets were kept at −20 °C. Total RNA was extracted from cell pellets using as RNeasy Mini Kit (Promega) as recommended by the manufacturer. The RNA was reversed transcribed using random primers (M-MLV Reverse Transcriptase kit, Promega) according to the manufacturer’s instructions. Gene expression was then assayed by real-time PCR using Kapa Syber Fast (Kapa Biosystems) on a Rotor-Gene Q cycler (Qiagen) system. Beta-actin primers (forward—GGCTGTATTCCCCTCCATCG, reverse—CCAGTTGGTAACAATGCCTGT) were used for the housekeeping gene, and Axin2 primers (forward—TGACTCTCCTTCCAGATCCCA, reverse—TGCCCACACTAGGCTGACA) were used for the read-out of Wnt/β-catenin activity. Reactions were performed in triplicate, and relative changes to housekeeping gene were calculated by the ΔΔ*C*_T_ method.

### Elemental analysis by inductively coupled plasma mass spectroscopy (ICP–MS)

ICP–MS was carried to assess the concentration of calcium, lithium, phosphorus and silicon ions in cell culture media. Solutions were diluted 1:100 in 1% nitric acid and analysed on a Perkin Elmer NexION 350D with a CETAC AX520 autosampler, using customary calibration standards. Data were analysed using Syngistix software.

### Statistical analyses

All data are presented as means + standard deviation and represent data from at least three independent experiments. Statistical analyses were carried out using one-way analysis of variance followed by post hoc Tukey test. Differences were considered significant if *p* < 0.05.

## Results and discussion

**Figure 1:**
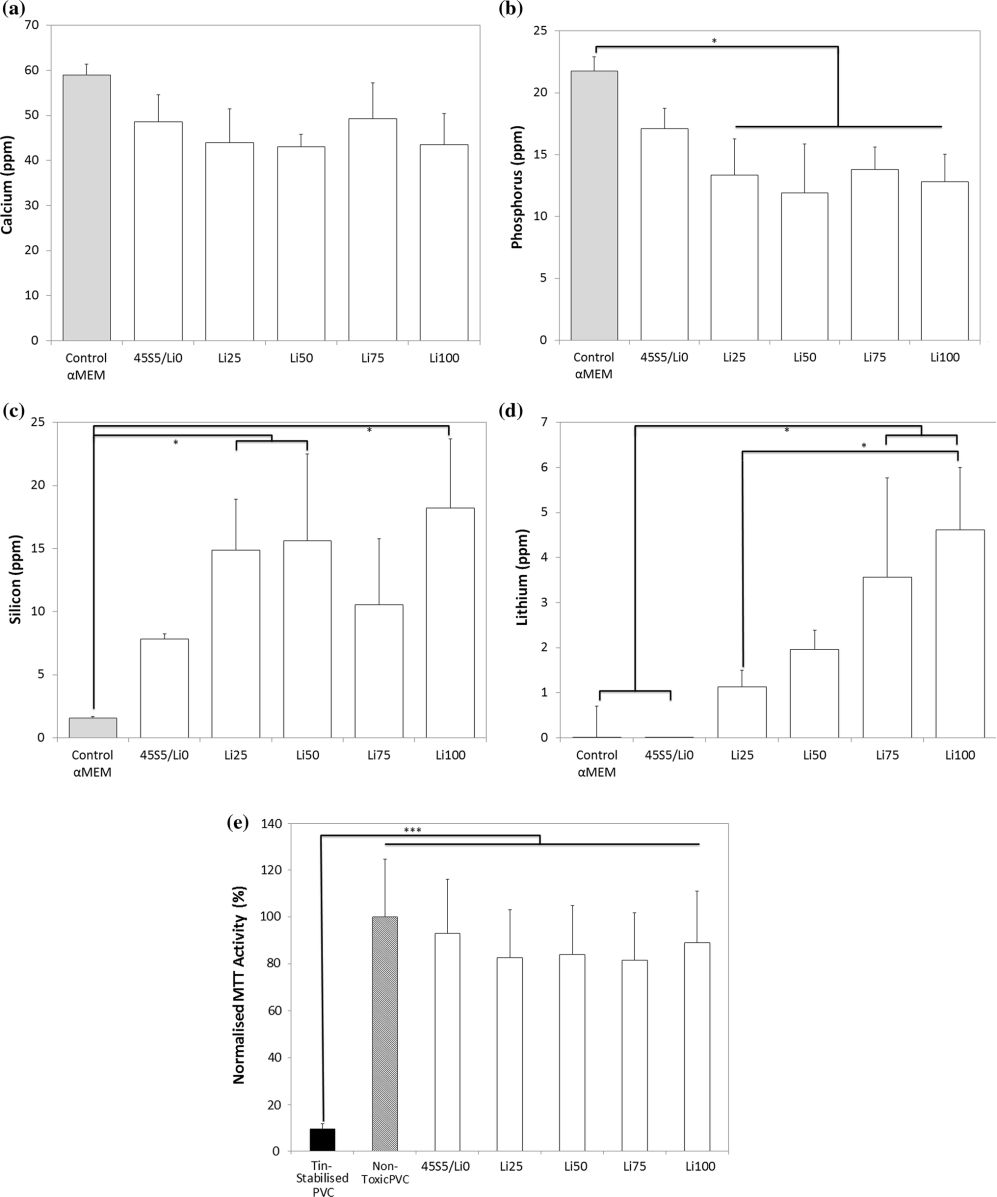

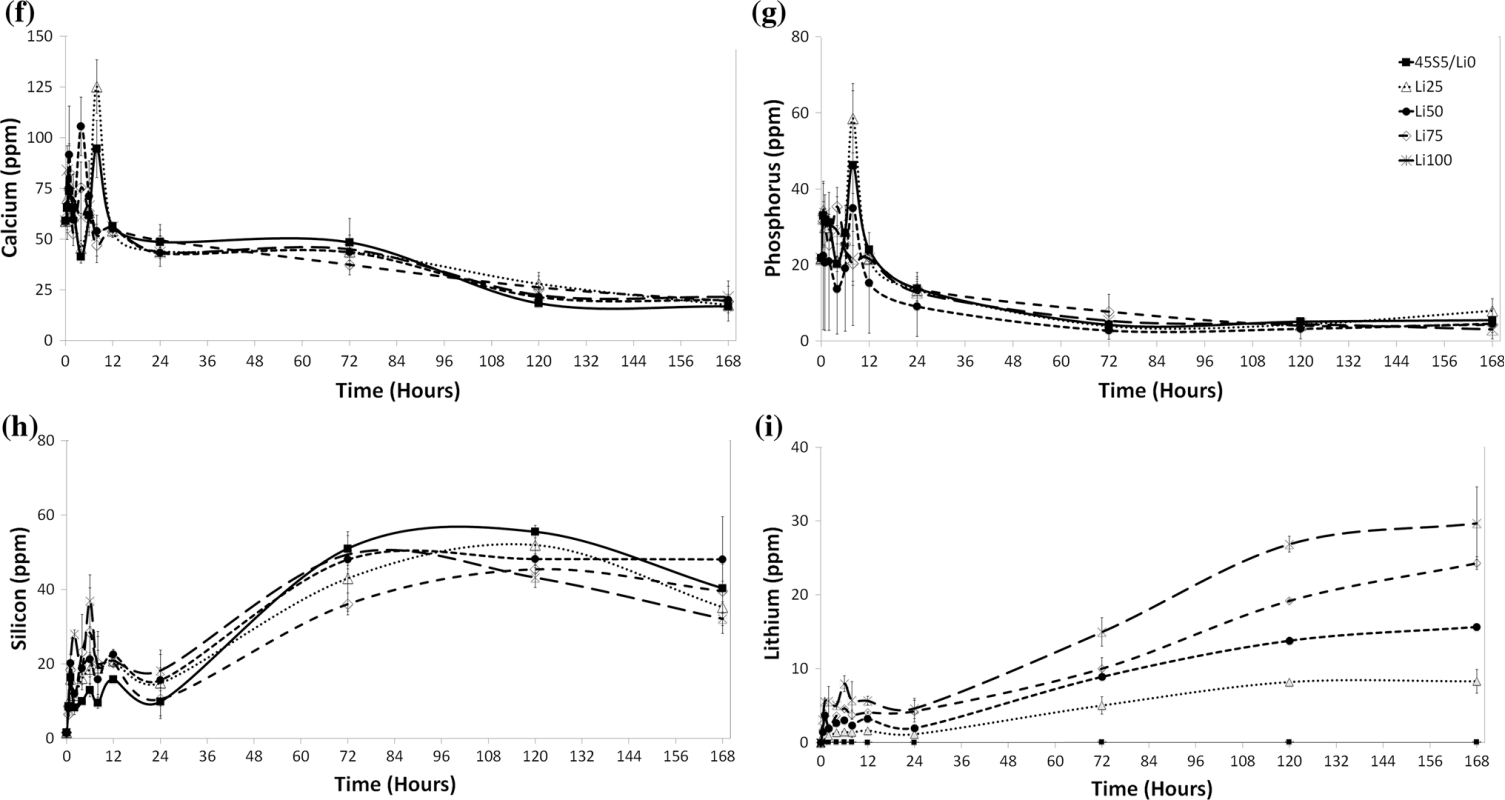
Ionic concentration of **a** calcium, **b** phosphorus, **c** silicon and **d** lithium in cell culture media after 24-h soaking with 6 mg/mL (1×) large BG particles. **e** Normalised metabolic activity of MC3T3-E1 cells after 24-h treatment with 1× (large particles) BG dissolution medium. Ionic concentration of **f** calcium, **g** phosphorus, **h** silicon and **i** lithium in cell culture media after 7-day soaking with 6 mg/mL (1×) large BG particles. *p < 0.05; ***p < 0.001

We first examined the ionic composition of cell culture medium after soaking with large (0.1–1 mm) BG particles at the 1× concentration (6 mg/mL) for 24 h (Fig. 1a–d). The concentration of calcium in cell culture medium was similar to that in controls not soaked with BG particles; whilst that of phosphorus was significantly lower in the Li25, Li50, Li75 and Li100 groups compared to controls. The concentration of silicon in culture media varied based on BG composition, with Li25, Li50 and Li100 groups containing significantly higher concentrations than controls. The concentration of lithium in cell culture media increased with increasing substitution of lithium into the BG, and the differences in the Li75 and Li100 groups were significantly different from that of 45S5/Li0 controls. The metabolic activities of MC3T3-E1 cells treated with dissolution media were all similar to that of the negative control and were significantly different than that of the positive (cytotoxic) control (Fig. 1e), confirming their lack of toxicity.

To determine the effect of time on ion release into cell culture medium, we extended the incubation time up to 7 days (Fig. 1f–i). Whilst calcium and phosphorus concentrations decreased and silicon increased, with time in all groups, ICP–MS analysis confirmed that the concentration of lithium in the medium of all groups soaked with lithium-containing BG increased with time and continued to be governed by the amount of lithium substituted into the BG.

Ion release patterns observed here differ markedly from those observed previously in Tris buffer solution [24], in which ion release occurs more quickly. That is, in Tris buffer 60% of lithium ions are released within the first 24 h [24, 29]. Ions such as calcium, sodium and lithium are released from BG in exchange for protons (H^+^ ions) from the immersion medium [6], and previous studies have shown marked differences in ion release and calcium phosphate surface layer formation from BG immersed in Tris buffer [30] compared to that in simulated body fluid [31] and cell culture medium [7]. Whilst cell culture medium contains relatively high concentrations of calcium and sodium (similar to SBF, which is close to saturation in regard to apatite [32]), Tris buffer does not, which may account for these differences. We also examined ion release in culture medium prior to adding serum. The presence of proteins has been shown to affect ion release from, and apatite formation on BG [33, 34], but should not have played a role here.

**Figure 2:**
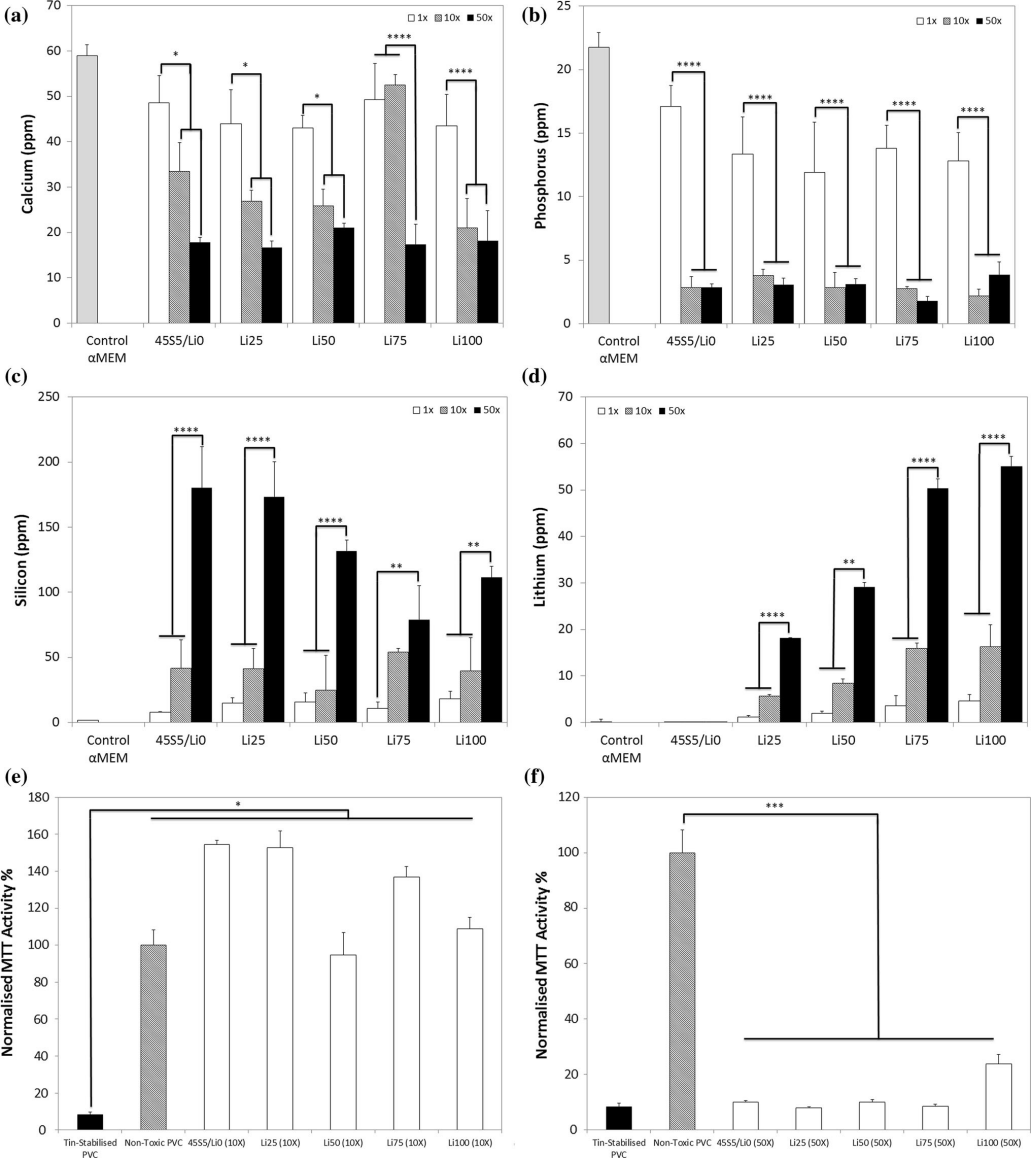

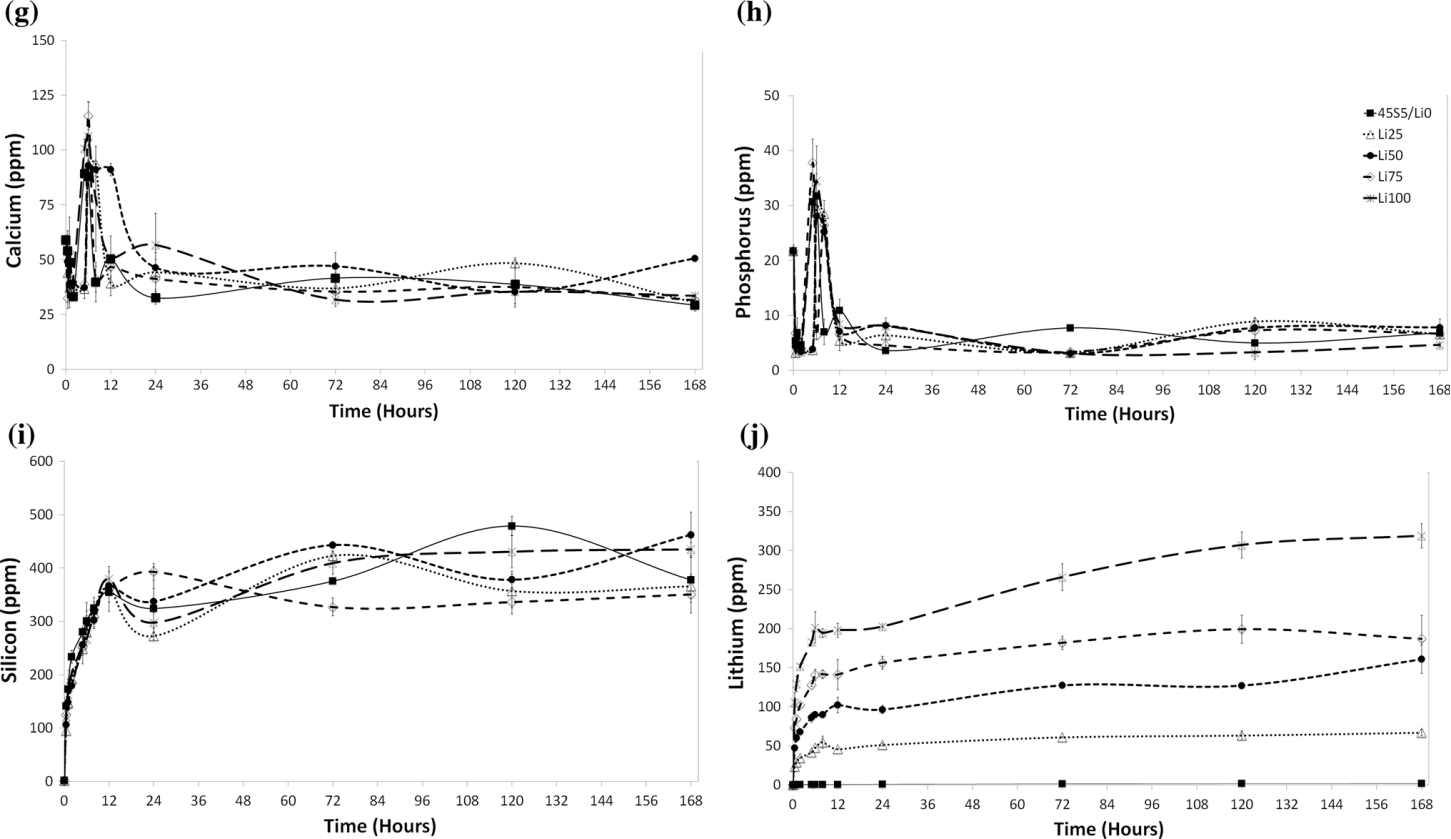
Ionic concentration of **a** calcium, **b** phosphorus, **c** silicon and **d** lithium in cell culture media after 24-h soaking with 6 mg/mL (1×), 60 mg/mL (10×) or 300 mg/mL (50×) large BG particles. **e** Normalised metabolic activity of MC3T3-E1 cells after 24-h treatment with 10× (large particles) BG dissolution medium. **f** Normalised metabolic activity after 24-h treatment with 50× (large particles) BG dissolution medium. Ionic concentration of **g** calcium, **h** phosphorus, **i** silicon and **j** lithium in cell culture media after 7-day soaking with 60 mg/mL (10×) small BG particles. *p < 0.05; **p < 0.01; ***p < 0.001; ****p < 0.0001

To determine the effect of the concentration of BG particles in the solution, we next examined ion release after increasing the mass of BG in the same volume of medium by factors of 10 and 50. After 24 h, the concentration of calcium in the medium of all groups (except Li75) was significantly lower in the 10× and 50× groups compared to the 1× groups (Fig. 2a). Similarly, the concentration of phosphorus in the medium was significantly lower with increasing concentration of BG particles (Fig. 2b). This result seems counterintuitive as one would expect higher concentrations of BG to result in increased ion release. And indeed, the release of lithium from all BG followed the expected trend of increased lithium release with increasing concentration of BG particles. However, as both calcium and phosphate are key components of apatite, a mineral phase in which BG are known to form on their surface when in contact with physiological solutions [35], and as calcium and phosphorus depletion is observed commonly during apatite formation of BG [36], this observation suggests that apatite formation was enhanced with increasing BG concentration, resulting in faster calcium and phosphorus depletion from the culture medium. Lithium, by contrast, is a typical glass modifier [6], and therefore is easily released from BG by ion exchange. Upon release, it is incorporated into apatite in small amounts only [37], if at all, and concentrations in solution therefore depend on release from the BG and are not affected by precipitation reactions. Indeed, lithium concentrations in solution seem to depend on factors including substitution in the BG, BG concentration in solution as well as immersion medium composition.

The release of silicon showed a more complex trend. Whilst increasing the concentration of BG particles increased medium silicon concentrations in all groups (except Li75, Fig. 2c), BG composition also had an effect with a trend for decreasing silicon release with increasing substitution of lithium for sodium in the BG (50× only). This trend likely arises from the effect lithium for sodium substitution has on the BG silicate network. As lithium has a smaller ionic radius than sodium, BG dissolution is reduced [24], which will diminish the expected increase in solution pH. Therefore, as both alkaline hydrolysis of the silicate network and the solubility limit of silicon species in aqueous solution are known to be highly pH dependent [24], the release of silicon is likely similarly reduced. It is well documented that silicon ions affect cell response, particularly within the osteogenic lineage [38]. Therefore, as its ion concentration in solution changes concomitant with changes in lithium and calcium ion concentrations, it is difficult to definitively attribute changes in cell response to the presence of one ion or another.

To determine the toxicity of the dissolution media after increasing BG concentration, we again measured cell metabolic activity according to ISO10993:5 tests. The normalised MTT activities of cells treated with 10× media were all significantly higher than that of positive controls (Fig. 2e), and none were significantly lower than that of negative controls, confirming their lack of toxicity. Cells treated with 50× BG, however, showed significantly lower metabolic activity than negative controls, suggesting these compositions at these concentrations were toxic to cells (Fig. 2f).

To determine the effect of increasing BG surface area on ion release, we next created smaller particles (<38 µm) and analysed ion release at the 10× concentration over 7 days. Similar to trends with larger BG particles, we observed decreases in the concentrations of calcium and phosphorus in the media with decreasing average particle size (i.e. increasing relative surface area) compared to controls (Fig. 2g, h). This, again, is likely attributable to faster ion release and subsequent faster apatite formation with increasing surface area. Similarly, the concentration of silicon increased with time in all groups (Fig. 2i) and the levels of lithium followed expected trends with increases in time and with increasing lithium content in the BG (Fig. 2j). Nevertheless, the overall concentrations of lithium were in the range of one order of magnitude higher compared to that achieved with larger particles.

Ion release patterns for smaller particles resemble more closely those observed in previous studies in Tris buffer [24], with fast increases in ion concentrations over the first 12 h, followed by relatively constant concentrations over the remaining time of the experiment. As previous studies were also performed using small (<38 µm) BG particles, and as ions are released in an exchange mechanism at the BG surface, this suggests a strong role for the surface area to volume ratio in addition to that of immersion medium composition in affecting ion release. In short, in in vitro studies, BG particle size as well as culture medium composition needs to be accounted for when evaluating ion release.

**Figure 3:**
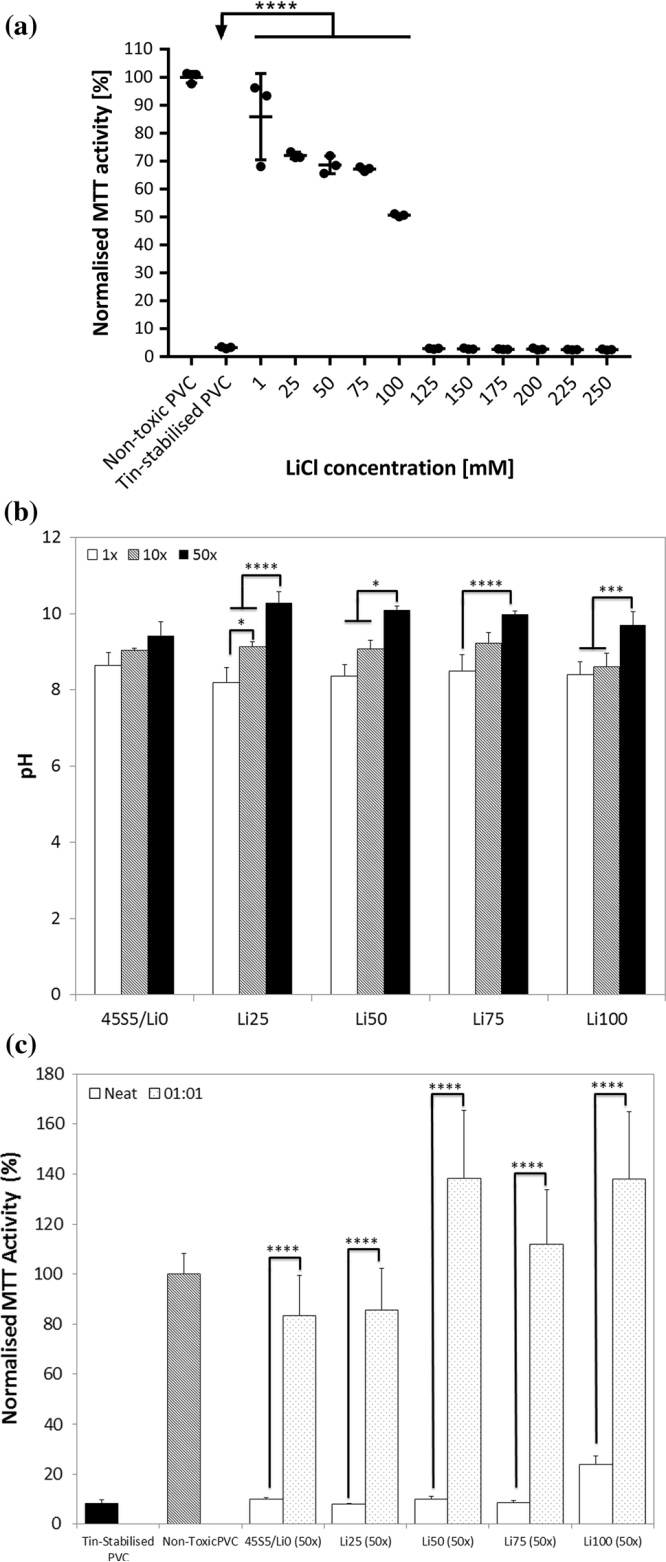
**a** Normalised metabolic activity of MC3T3-E1 cells after treatment with increasing concentrations of LiCl. **b** pH of cell culture media after soaking for 24 h with 1×, 10× and 50× concentrations of large BG particles. **c** Normalised metabolic activity of MC3T3-E1 cells after 24-h treatment with 50× large particles neat and diluted 1:1 with fresh cell culture medium. *p < 0.05; ***p < 0.001; ****p < 0.0001

As toxicity of dissolution medium was high in cells treated with 50× medium, which in some cases released relative high amounts of lithium compared to 1× and 10× groups, we hypothesised that toxicity may have been mediated by the presence of lithium ions. To test this, we measured the metabolic activity of cells exposed to increasing concentrations of LiCl (Fig. 3a). Metabolic activity of cells exposed to LiCl was significantly different from that of positive controls at concentrations up to 100 mM. At higher concentrations, toxicity was evident as metabolic activity was significantly different to that of negative controls and similar to that of cells treated with medium from toxic positive control materials.

As lithium itself was not toxic at concentrations more than an order of magnitude higher than those detected in dissolution medium, we next hypothesised that toxicity was mediated by changes in solution pH. We measured the pH of dissolution medium formed with 1×, 10× and 50× concentrations of BG (large particles) and found that the pH of 50× lithium-containing BG treated media was significantly higher than that of 1× controls (Fig. 3b). Although cell culture medium is buffered, previous reports have similarly shown that BG can cause a cytotoxic rise in pH [39]. Mechanistically this arises as a surplus of hydroxyl (OH^−^) groups is formed from the ion exchange between modifier cations (such as calcium, sodium or lithium ions) from the BG and protons from the solution. Diluting these media 1:1 with fresh cell culture media resulted in significant increases in cell metabolic activities that were similar to those of negative controls (Fig. 3c), lending further support to our pH hypothesis.

**Figure 4:**
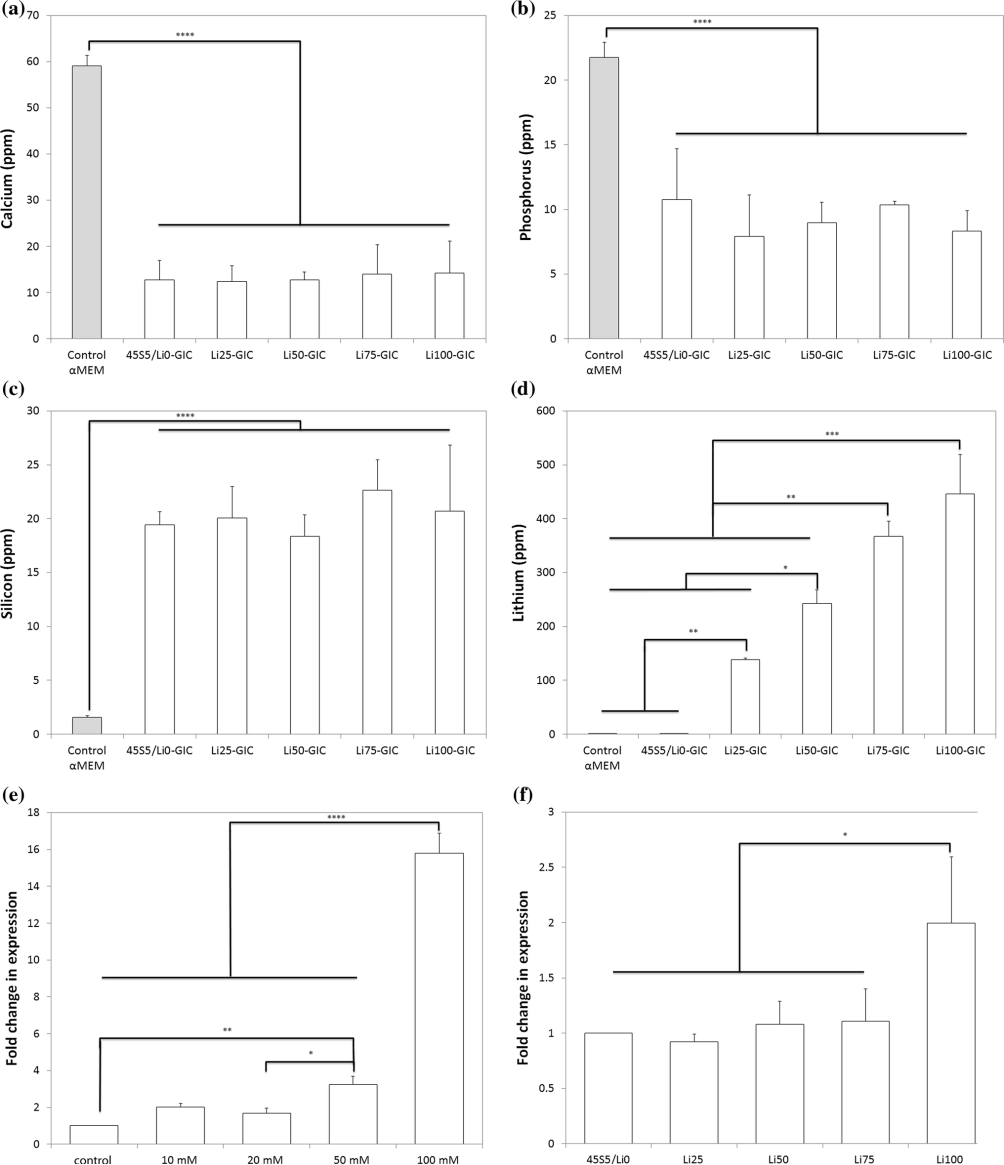
Concentration of **a** calcium, **b** phosphorus, **c** silicon and **d** lithium in cell culture medium after soaking for 7 days with BG-modified GIC according to ISO10993:5. **e** Fold change in expression of *Axin2* relative to vehicle controls in 17IA4 cells after 24-h treatment with varying concentrations of LiCl. **f** Fold change in expression of *Axin2* relative to vehicle controls in 17IA4 cells after 24-h treatment with 1× large BG particles soaked in culture medium for 24 h. *p < 0.05; **p < 0.01; ***p < 0.001; ****p < 0.0001

We next asked whether we could tailor GIC to also release therapeutic levels of lithium. To achieve this, we substituted some of the powder phase of a commercial GIC with lithium-substituted BG. All BG compositions allowed for the formation of stable cements, confirming that the substitution did not have a dramatic effect on mixing, setting or mechanical stability (as determined by visual observation). To determine ion release, we soaked the cements in cell culture media according to ISO10993:5 and measured ion release by ICP–MS (Fig. 4a–d). Calcium and phosphorus levels in the media were significantly lower in GIC-treated groups compared to standard culture medium, and the concentration of silicon was significantly higher. Lithium release from the GIC followed expected trends with significant increases in lithium in the medium with increasing concentrations of lithium in the BG. Lithium-releasing GIC have the potential to be used in applications such as tooth regeneration and/or maintenance, in which Wnt/β-catenin signalling plays an important role [40].

Having demonstrated our ability to tailor lithium release from the BG and GIC over a broad, sub-toxic range via lithium substitution in the BG, we next asked what level of lithium would need to be released to target a biological response, specifically activation of the Wnt/β-catenin pathway. The concentration of lithium in the serum of patients on lithium maintenance therapy has been reported to be 0.8 mmol/L (5.5 ppm) [20], so this range has been suggested to be therapeutic. However, other studies have suggested that much higher levels (e.g. 20 mM, 139 ppm) are necessary to upregulate the Wnt pathway [19]. To determine levels necessary to induce a biological response, we exposed 17IA4 mouse dental pulp cells to increasing (but sub-toxic) concentrations of LiCl and carried out quantitative analysis of expression of the gene *Axin2*, which plays important roles in the regulation of β-catenin in the Wnt pathway. We observed a dose-dependent increase in *Axin2* expression with increasing LiCl concentrations between 10 and 50 mM (Fig. 4e). Treatment of 17IA4 cells with 100 mM LiCl (694 ppm) stimulated an approximately 15-fold increase in expression compared to vehicle controls. To confirm that similar responses could be elicited by lithium released from BG, we treated 17IA4 cells with 1× media (24 h, large particles), which contains concentrations of lithium as reported in Fig. 1d. We observed significantly higher expression of *Axin2* in cells treated with Li100 compared to 45S5/Li0 and all other groups (Fig. 4f).

## Conclusions

Here we show that we can tailor the release of lithium from BG over a broad range by adjusting BG composition, concentration in cell culture medium and particle size. The resulting dissolution medium is non-toxic for many of the combinations of parameters we tested and can elicit an effect on Wnt/β-catenin signalling that mirrors that induced by treating cells with LiCl. High concentrations of BG in cell culture media can induce a cytotoxic response that is likely mediated by changes in solution pH, but this can be ameliorated by diluting dissolution medium with fresh medium or using a lower concentration of the BG. These studies may be important in the design of non-toxic lithium-releasing materials that can modulate the Wnt pathway for hard tissue regeneration.

## References

[1] Jones JR, Gentleman E, Polak J (2007). Bioactive glass scaffolds for bone regeneration. Elements.

[2] Gentleman E, Polak JM (2006). Historic and current strategies in bone tissue engineering: do we have a hope in Hench?. J Mater Sci Mater Med.

[3] Hoppe A, Guldal NS, Boccaccini AR (2011). A review of the biological response to ionic dissolution products from bioactive glasses and glass-ceramics. Biomaterials.

[4] Cattalini JP, Hoppe A, Pishbin F (2015). Novel nanocomposite biomaterials with controlled copper/calcium release capability for bone tissue engineering multifunctional scaffolds. J R Soc Interface.

[5] Valerio P, Pereira MM, Goes AM, Leite MF (2004). The effect of ionic products from bioactive glass dissolution on osteoblast proliferation and collagen production. Biomaterials.

[6] Brauer DS (2015). Bioactive glasses—structure and properties. Angew Chem Int Ed.

[7] Gentleman E, Stevens MM, Hill RG, Brauer DS (2013). Surface properties and ion release from fluoride-containing bioactive glasses promote osteoblast differentiation and mineralization in vitro. Acta Biomater.

[8] Azevedo MM, Tsigkou O, Nair R, Jones JR, Jell G, Stevens MM (2015). Hypoxia inducible factor-stabilizing bioactive glasses for directing mesenchymal stem cell behavior. Tissue Eng Part A.

[9] Gentleman E, Fredholm YC, Jell G (2010). The effects of strontium-substituted bioactive glasses on osteoblasts and osteoclasts in vitro. Biomaterials.

[10] O’Donnell MD, Candarlioglu PL, Miller CA, Gentleman E, Stevens MM (2010). Materials characterisation and cytotoxic assessment of strontium-substituted bioactive glasses for bone regeneration. J Mater Chem.

[11] Autefage H, Gentleman E, Littmann E (2015). Sparse feature selection methods identify unexpected global cellular response to strontium-containing materials. Proc Natl Acad Sci USA.

[12] Fuchs M, Gentleman E, Shahid S, Hill R, Brauer D (2015). Therapeutic ion-releasing bioactive glass ionomer cements with improved mechanical strength and radiopacity. Front Mater.

[13] Brauer DS, Karpukhina N, Kedia G (2013). Bactericidal strontium-releasing injectable bone cements based on bioactive glasses. J R Soc Interface.

[14] Miola M, Fucale G, Maina G, Verne E (2015). Antibacterial and bioactive composite bone cements containing surface silver-doped glass particles. Biomed Mater.

[15] Brauer DS, Gentleman E, Farrar DF, Stevens MM, Hill RG (2011). Benefits and drawbacks of zinc in glass ionomer bone cements. Biomed Mater.

[16] Klein PS, Melton DA (1996). A molecular mechanism for the effect of lithium on development. Proc Natl Acad Sci USA.

[17] Day TF, Guo XZ, Garrett-Beal L, Yang YZ (2005). Wnt/β-catenin signaling in mesenchymal progenitors controls osteoblast and chondrocyte differentiation during vertebrate skeletogenesis. Dev Cell.

[18] Sarkar L, Sharpe PT (1999). Expression of Wnt signalling pathway genes during tooth development. Mech Dev.

[19] Clement-Lacroix P, Ai M, Morvan F (2005). Lrp5-independent activation of Wnt signaling by lithium chloride increases bone formation and bone mass in mice. Proc Natl Acad Sci USA.

[20] Zamani A, Omrani GR, Nasab MM (2009). Lithium’s effect on bone mineral density. Bone.

[21] Ishimoto K, Hayano S, Yanagita T (2015). Topical application of lithium chloride on the pulp induces dentin regeneration. PLoS One.

[22] Shan TF, Zhou C, Yang R (2015). Lithium chloride promotes the odontoblast differentiation of hair follicle neural crest cells by activating Wnt/beta-catenin signaling. Cell Biol Int.

[23] Tylkowski M, Brauer DS (2013). Mixed alkali effects in Bioglass^®^ 45S5. J Noncryst Solids.

[24] Bruckner R, Tylkowski M, Hupa L, Brauer DS (2016). Controlling the ion release from mixed alkali bioactive glasses by varying modifier ionic radii and molar volume. J Mater Chem B.

[25] Miguez-Pacheco V, Buttner T, Macon ALB (2016). Development and characterization of lithium-releasing silicate bioactive glasses and their scaffolds for bone repair. J Noncryst Solids.

[26] Khorami M, Hesaraki S, Behnamghader A, Nazarian H, Shahrabi S (2011). In vitro bioactivity and biocompatibility of lithium substituted 45S5 bioglass. Mater Sci Eng C.

[27] Han PP, Wu CT, Chang J, Xiao Y (2012). The cementogenic differentiation of periodontal ligament cells via the activation of Wnt/beta-catenin signalling pathway by Li^+^ ions released from bioactive scaffolds. Biomaterials.

[28] Keller L, Kuchler-Bopp S, Mendoza SA, Poliard A, Lesot H (2011). Tooth engineering: searching for dental mesenchymal cells sources. Front Physiol.

[29] Brauer DS, Bruckner R, Tylkowski M, Hupa L (2016). Sodium-free mixed alkali bioactive glasses. Biomed Glasses.

[30] Brauer DS, Mneimne M, Hill RG (2011). Fluoride-containing bioactive glasses: fluoride loss during melting and ion release in tris buffer solution. J Noncryst Solids.

[31] Brauer DS, Karpukhina N, O’Donnell MD, Law RV, Hill RG (2010). Fluoride-containing bioactive glasses: effect of glass design and structure on degradation, pH and apatite formation in simulated body fluid. Acta Biomater.

[32] Bohner M, Lemaitre J (2009). Can bioactivity be tested in vitro with SBF solution?. Biomaterials.

[33] Shah FA, Brauer DS, Wilson RM, Hill RG, Hing KA (2014). Influence of cell culture medium composition on in vitro dissolution behavior of a fluoride-containing bioactive glass. J Biomed Mater Res Part A.

[34] Shah FA, Brauer DS, Hill RG, Hing KA (2015). Apatite formation of bioactive glasses is enhanced by low additions of fluoride but delayed in the presence of serum proteins. Mater Lett.

[35] Jones JR (2013). Review of bioactive glass: from Hench to hybrids. Acta Biomater.

[36] Bingel L, Groh D, Karpukhina N, Brauer DS (2015). Influence of dissolution medium pH on ion release and apatite formation of Bioglass^®^ 45S5. Mater Lett.

[37] Elliot JC (1994). Structure and chemistry of the apatites and other calcium orthophosphates.

[38] Shie MY, Ding SJ, Chang HC (2011). The role of silicon in osteoblast-like cell proliferation and apoptosis. Acta Biomater.

[39] Wallace KE, Hill RG, Pembroke JT, Brown CJ, Hatton PV (1999). Influence of sodium oxide content on bioactive glass properties. J Mater Sci Mater Med.

[40] Lim WH, Liu B, Cheng D (2014). Wnt signaling regulates pulp volume and dentin thickness. J Bone Miner Res.

